# Sustaining Improvements of Surgical Site Infections by Six Sigma DMAIC Approach

**DOI:** 10.3390/healthcare10112291

**Published:** 2022-11-15

**Authors:** Zhi-Yuan Shi, Pei-Hsuan Huang, Ying-Chun Chen, Hui-Mei Huang, Yuh-Feng Chen, I-Chen Chen, Yi-Jing Sheen, Ching-Hui Shen, Jau-Shin Hon, Chin-Yin Huang

**Affiliations:** 1 Infection Control Center, Taichung Veterans General Hospital, Taichung 407219, Taiwan; 2 College of Medicine, National Chung Hsing University, Taichung 40227, Taiwan; 3 Department of Industrial Engineering & Enterprise Information, Tunghai University, Taichung 407224, Taiwan; 4 Nursing Department, Taichung Veterans General Hospital, Taichung 407219, Taiwan; 5 Department of Surgery, Taichung Veterans General Hospital, Taichung 407219, Taiwan; 6 Division of Endocrinology and Metabolism, Department of Internal Medicine, Taichung Veterans General Hospital, Taichung 407219, Taiwan; 7 Department of Anesthesiology, Taichung Veterans General Hospital, Taichung 407219, Taiwan; 8 School of Medicine, National Yang Ming Chiao Tung University, Taipei 112304, Taiwan

**Keywords:** surgical site infections, Six Sigma, DMAIC, improvement, antibiotic

## Abstract

Background: SSIs (surgical site infections) are associated with increased rates of morbidity and mortality. The traditional quality improvement strategies focusing on individual performance did not achieve sustainable improvement. This study aimed to implement the Six Sigma DMAIC method to reduce SSIs and to sustain improvements in surgical quality. The surgical procedures, clinical data, and surgical site infections were collected among 42,233 hospitalized surgical patients from 1 January 2019 to 31 December 2020. Following strengthening leadership and empowering a multidisciplinary SSI prevention team, DMAIC (Define, Measure, Analyze, Improve, and Control) was used as the performance improvement model. An evidence-based prevention bundle for reduction of SSI was adopted as performance measures. Environmental monitoring and antimicrobial stewardship programs were strengthened to prevent the transmission of multi-drug resistant microorganisms. Process change was integrated into a clinical pathway information system. Improvement cycles by corrective actions for the risk events of SSIs were implemented to ensure sustaining improvements. We have reached the targets of the prevention bundle elements in the post-intervention period in 2020. The carbapenem resistance rates of Enterobacteriaceae and *P. aeruginosa* were lower than 10%. A significant 22.2% decline in SSI rates has been achieved, from 0.9% for the pre-intervention period in 2019 to 0.7% for the post-intervention period in 2020 (*p* = 0.004). Application of the Six Sigma DMAIC approach could significantly reduce the SSI rates. It also could help hospital administrators and quality management personnel to create a culture of patient safety.

## 1. Introduction

The incidence of surgical site infections (SSIs) is estimated to be 2 to 5% in patients undergoing surgery in the USA [1]. In the annual report for 2017 from the 12 European Union countries and one European Economic Area country, the cumulative incidence of patients with SSI was the highest in open colon surgery with 10.1 SSIs per 100 operations (10.1%), followed by 6.4% for laparoscopic colon surgery, 3.9% for open cholecystectomy, 2.6% for coronary artery bypass graft, 1.8% for caesarean section, 1.5% laparoscopic cholecystectomy, for 1.0% for hip prosthesis, 0.8% for laminectomy, and 0.5% for knee prosthesis [2]. The pooled SSI rate in low- and middle-income countries was 11.2% (95% CI: 9.7–12.8) for incidence/prospective studies from an updated systemic literature review from 1995 to 2015 conducted by WHO [3]. SSIs are associated with a longer hospital stay and increased rates of morbidity and mortality [4]. The financial cost of SSIs is the highest among all the healthcare-associated infections (HAIs) [5]. The annual cost of SSIs in the USA has been estimated to be $3.5 to $10 billion [3]. About 40% of SSIs are preventable by the interventions of evidence-based measures [1,3,6].

The National Surgical Infection Prevention Project (SIP) was conducted by 56 USA hospitals in 2002 [7]. This project demonstrated a 27% reduction of SSIs by a quality improvement approach with five key measures, including appropriate antimicrobial agent selection, timing, and duration, normothermia, oxygenation, controlling blood glucose, and appropriate hair removal. SIP only focused on a limited set of surgical procedures, such as elective orthopedic or colorectal procedures, or all coronary artery bypass graft [7]. SIP was expanded to the Surgical Care Improvement Project (SCIP) in 2006, with SSI prevention measures and additional recommendations for the prevention of cardiac adverse events and venous thromboembolism [8]. The risk factors of SSIs are complex and multi-factorial. The patient-related risk factors include diabetes mellitus, old age, chronic systemic disease, and colonized resistant organisms. The treatment-related factors include surgical techniques, type of surgical procedures, duration of surgical procedures, prosthesis/implants, and the measures in SCIP [3,8]. However, in a systemic review, the effects of SCIP measures only demonstrated a cumulative 4% decrease in SSIs [8]. It is apparent that improvement in the compliance of individual SCIP measures alone is unlikely to result in effective reduction of SSI rates [9,10]. In contrast, the improvement projects using systematic approaches, or care bundles that incorporate best practices for the perioperative care, have been successful in reducing SSI rates to varying degrees [9,11]. A meta-analysis has indicated that the effects of perioperative care bundles for the prevention of SSIs are inconsistent among many randomized control trials [12]. Prevention bundle will not result in decreased SSI rates, if the overall compliance or systemic adaptation by healthcare organizations is insufficient [13,14]. Therefore, a more comprehensive quality improvement approach is needed to achieve the goal of reduction in SSIs [13,14,15,16].

The Six Sigma management tools used in business processes have been applied to the quality control of healthcare processes. Six Sigma uses a powerful framework (DMAIC: Define, Measure, Analyze, Improve and Control) and statistical tools to identify root causes of deviations from the ideal process, to improve process performance by addressing and eliminating the defects, and to control the improved process and sustain gains. Six Sigma has been implemented in the healthcare setting for process improvement to decrease errors, improve patient satisfaction, and reduce length of stay. Montella et al. applied Lean Six Sigma methodology to reduce the risks of healthcare-associated infections in surgery departments, thereby reducing the percentage of colonized patients from 0.37% to 0.21% and reducing the average length of hospital stay from 45 to 36 days in surgical departments [17]. Improta et al. applied corrective actions to reduce the average length of stay from 18.9 to 10.6 days (a reduction of 44%) for patients undergoing prosthetic hip replacement surgery in an Italian hospital [18]. Tagge et al. improved operating room efficiency at a US children’s hospital. The interval between a patient leaving the operating room and the arrival of the next one decreased from a median time of 41 min to 32 min, and the interval between dressing and the subsequent surgical incision decreased from 81.5 min to 71 min [19].

Only limited studies focused on reducing HAIs. Kuwaiti et al. reduced the HAI rate (from HAI rate of 3.92% during the preintervention phase to 2.73% during the postintervention phase) [15]. Cesarelli et al., dealt with HAIs at an Italian rehabilitation hospital, and the overall decrease in HAI rates in the hospital was 3.4% (from 15.3% to 11.9%) [20].

Hospital environments are highly susceptible to bacterial contamination with antibiotic-resistant organisms such as methicillin-resistant *Staphylococcus aureus*, vancomycin-resistant enterococci (VRE), Enterobacteriaceae, *Acinetobacter* spp., and *Pseudomonas aeruginosa*. These contaminated environmental surfaces may contribute to the transmission of pathogens and subsequent HAIs [21]. Environmental cleaning and monitoring is an important part of strategy to reduce the risk of HAIs [21]. An antimicrobial stewardship program has been demonstrated to reduce the incidence of colonization with antibiotic-resistant bacteria and infection in hospitalized patients [22]. Multi-drug resistant organisms are emerging as a significant cause of surgical site infections [23], therefore, the strategy of reducing the incidence of MDRO in SSI needs to be analyzed.

The aim of the present study was to apply DMAIC methodology to reduce SSIs by identifying and improving the variables that increase the risks of SSIs. In this approach, healthcare professionals (surgeons, nurses, operating department personnel, anesthesiologists, diabetes specialists, managers of general affairs office, pharmacists, and laboratory technicians, engineers of Information technology, and infection control practitioners) were organized and instructed to be accountable for the improvement of process and were able to analyze and solve problems efficiently and effectively. They implemented useful strategies to monitor and improve the performance measures, to standardize the surgical procedures and prevent the risks of SSIs, to prevent the transmission of multi-drug resistant microorganisms by environmental monitoring and antimicrobial stewardship, thereby aiming to reduce the SSI rates.

## 2. Methods

### 2.1. Design and Setting

**Figure 1:**
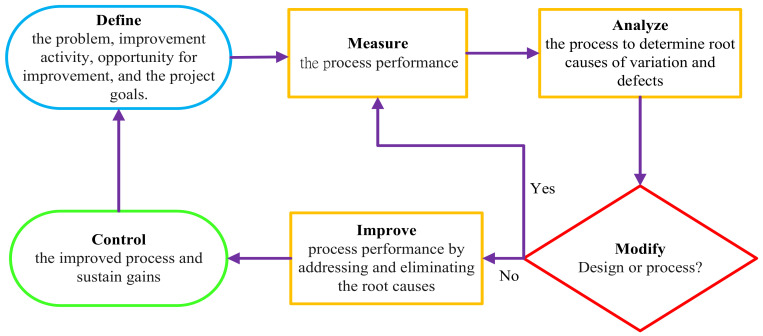
The visual diagram for the Six Sigma DMAIC structure.

The SSI rate at Taichung Veteran General Hospital was 1.2% (256 SSI events in 21,042 surgical operations) in 2018 and ranked as the 75th percentile in Taiwan. The historic data of this hospital’s SSI rates in 2018 were considered as the reference point. Therefore, a sustaining quality improvement project of SSIs was conducted at this hospital. The SSI rates during the study period of 2019 to 2020 were determined by the hospital’s infection control practitioners. The Six Sigma DMAIC (Define, Measure, Analyze, Improve, and Control model) approach was implemented during the study period, and its effectiveness to reduce the SSI rates was evaluated [24]. DMAIC refers to a cycle of process improvement that is data-driven and aims at identifying the defects, optimizing the designs and process, improving the outcome, and sustaining improvement. The five steps of DMAIC are outlined as the following processes: 1. Define the problem, improvement activity, opportunity for improvement, and the project goals; 2. Measure process performance to objectively establish current baseline performance; 3. Analyze the process to determine root causes of variation or defects; 4. Improve process performance by addressing and eliminating the root causes; 5. Control the improved process and sustain gains [24]. The visual diagram explaining the Six Sigma DMAIC structure is shown in Figure 1.

### 2.2. Define: Identifying the Study

The define phase started with a clear definition of the project goal, i.e., to reduce the SSI rates, and a multidisciplinary team (SSI prevention team) was organized to be responsible for the implementation of strategy to reduce SSI rates. The team leader was the dean of the Taichung Veterans General Hospital. The team members consisted of physicians (surgeons, diabetes specialists, anesthesiologists, and infectious disease specialists), supervisors of the nursing department, pharmacists, laboratory technicians, quality control practitioners, infection control practitioners, managers of the general affairs office, and information technology engineers. They report the improvement strategies and outcomes at the Infection Control Committee quarterly.

The SSI rate was defined as the number of SSI events per 100 operations, i.e., calculated by dividing the number of SSI events by the number of operative procedures and multiplying the results by 100 [25].

The surgical procedures, clinical data, and surgical site infections were collected for 42,233 hospitalized surgical patients from 1 January 2019 to 31 December 2020.

### 2.3. Measure: Data Collection

The study data were retrieved from the hospital medical information database. These data provide information concerning the independent variables of the process under investigation, including patients’ personal data (age and gender), patient hospitalization duration (days), surgical procedures, skin preparation, pre-operative shower, the timing and duration of prophylactic antibiotic, perioperative body temperature, blood glucose, laboratory data, diagnostic therapeutic procedures, SSI surveillance data, the isolates associated with SSIs, and the trend of antimicrobial resistance.

### 2.4. Analyse: Analysis of Causes

The Analyze phase was carried out by using Six Sigma management tools such as brainstorming and cause–effect diagrams. The risk events associated with surgical site infections were evaluated [3,6]. The objective of the Analyze stage was to find the root causes of risk events, so that they could be eliminated to improve the process. The team used a cause–effect diagram for the problem-solving processes. During the brainstorm process, SSI prevention team provided expert opinion to investigate the perioperative risks, protocols, procedures, and improvement strategies to reduce the risks associated with SSI.

### 2.5. Improve: Implement the Corrective Actions

After brainstorming and discussing the causes and problems in the Analyze phase, the SSI prevention team decided to identify and implement corrective actions aimed at improving the performance of the process.

### 2.6. Control: Implementation of the Control and Feedback System

The efficacy and efficiency of the implemented improvement actions were monitored over a 2-year period to investigate the effectiveness of the interventions aiming at the reduction of SSI rates. To continuously improve the process and maintain a high quality of surgical outcome, a quality control plan with healthcare information feedback system was implemented.

The continuous improvement plan was based on the DMAIC cyclic processes (Figure 1), reviewing the surgical outcomes regularly, brainstorming and discussing the causes and problems, identifying the defects, and implementing corrective actions aimed at improving the performance of the process.

We also emphasized the educational program for healthcare staff. The educational program is essential to make healthcare staff aware of risks and consequences of SSIs. The healthcare providers were also encouraged to be accountable and to adopt strategies to prevent SSIs.

**Figure 2:**
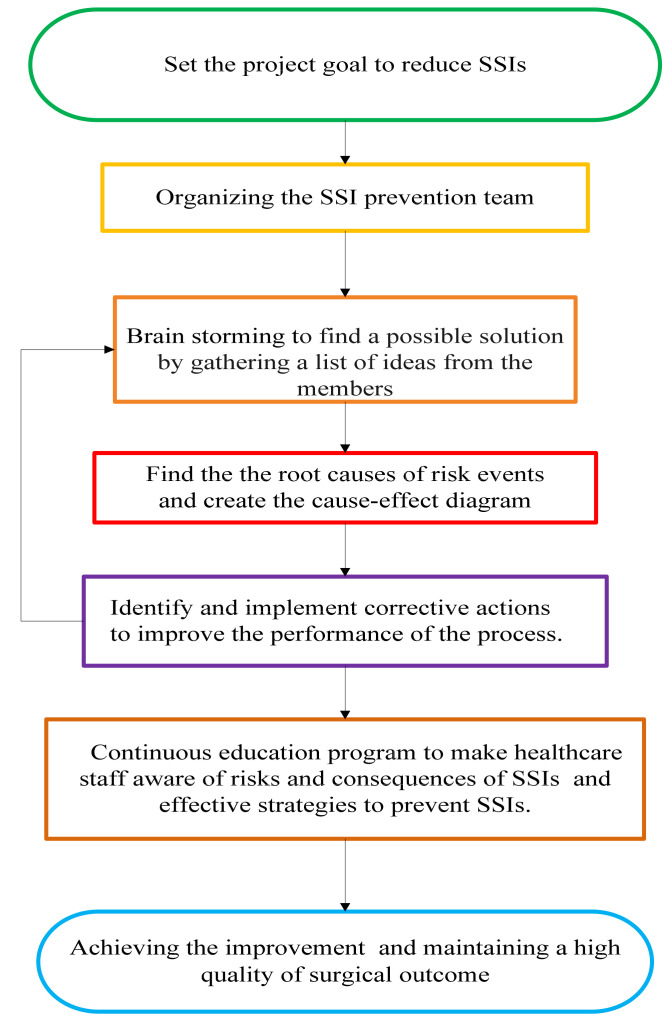
Flow map of the improvement process.

The flow map of the above improvement process is summarized and shown in Figure 2.

## 3. Results

### 3.1. Define

The hospital’s SSI rate was 1.2% in 2018 and ranked as the 75th percentile in Taiwan. The goal of reduction of the SSI rates was set at 20% by the end of 2020. Reduction of SSI rates is a continuous improvement process to meet the core value of zero tolerance of defects.

After literature review and discussion within the SSI prevention team, the goal of this project was defined as the reduction of SSI rates. The Six Sigma DMAIC project statement is shown in Table 1.

This phase was characterized by the development of the project statement, which clearly defined the problem, the goal for the reduction of SSI rates, organizing the members of the SSI prevention team, and the implementation of strategies to reduce SSI rates.

### 3.2. Measure

The study data were retrieved from the hospital’s medical information database. Five performance measures were proposed as the SSI prevention bundle and the indicators of quality improvement approach to reduce SSI [3,6,26,27,28]. The performance measures are listed as follows: (1) pre-operative shower or bath with soap or antiseptic agent; (2) appropriate hair removal method; (3) appropriate prophylactic antibiotic administration; (4) controlling blood glucose below 180 mg/dL preoperatively and during postoperative days 1 and 2; and (5) maintaining body temperature above 36 °C (normothermia) in the perioperative period.

SSI surveillance data, the SSI rates, the isolates associated with SSIs, and the trend of antimicrobial resistance were also collected for analysis.

### 3.3. Analyze

**Figure 3:**
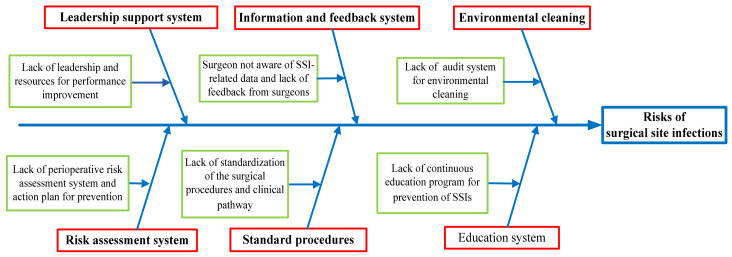
Cause–effect diagram of risk events associated with surgical site infections. The red boxes indicate categories of risks for surgical site infections. The green boxes indicate the main contributing causes for the categories.

During this phase, the data collected and measured in the Measure phase were analyzed. By reviewing the previous publications and brainstorming, a root cause analysis was carried out to determine the possible risk events associated with surgical site infections. A cause–effect diagram (Figure 3) was developed to identify the risk events and to provide corrective actions for process improvement.

The risk events associated with surgical site infections were classified into five different categories: (1) leadership support system to provide needed resources for performance improvement; (2) a perioperative risk assessment system to develop an infection prevention plan with measurable goals; (3) a healthcare information system to collect and monitor data of SSIs and to provide feedback to surgeons, (4) the standardization of surgical procedures and clinical pathways to reduce SSI; (5) continuous educational programs about the prevention and management of SSIs.

### 3.4. Improve

The SSI prevention team identified the risk events associated with surgical site infections and implement corrective actions aimed at improving the performance of surgical procedures and reducing the SSI rates. These corrective actions included strengthening leadership support systems, setting up perioperative risk assessment systems, standardizing surgical procedures and clinical pathways, designing automated data collection programs in healthcare information systems to collect SSI information and performance measures and then providing feedback to the surgeons, and providing educational programs (Table 2). The identified defects, improvement strategies, and outcomes were reported and reviewed at the Infection Control Committee quarterly.

**Figure 4:**
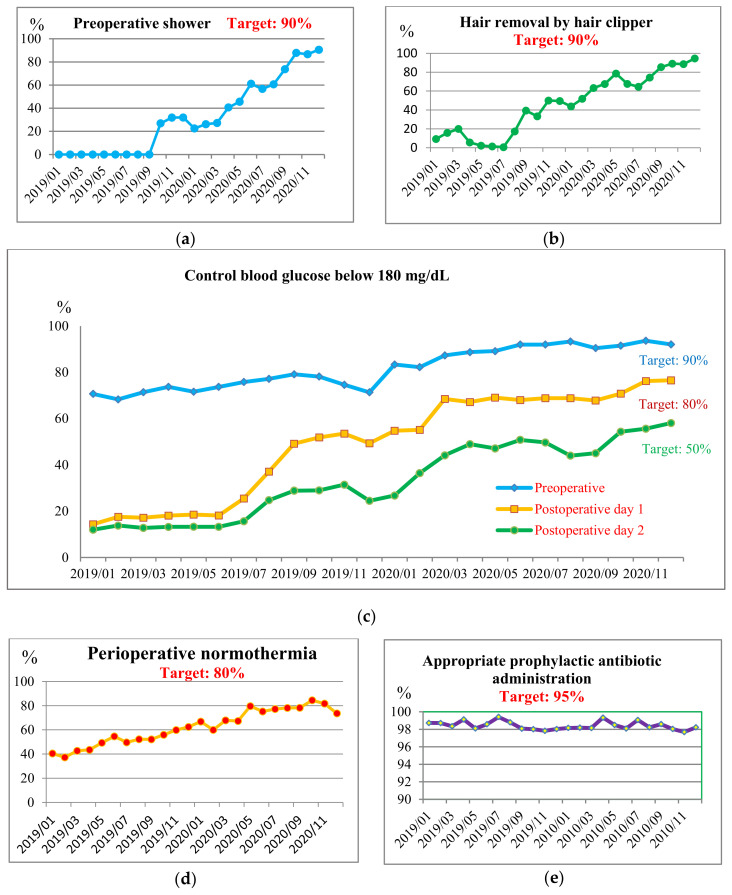
The improvement of performance measures of the 5 care bundle elements for SSI. (**a**) the percentage of preoperative shower; (**b**) skin preparation with hair clipper; (**c**) controlling blood glucose below 180 mg/dL; (**d**) maintaining perioperative normothermia; (**e**) appropriate prophylactic antibiotic administration. The targets are shown in the figures. The vertical axis indicates the performance measure of the prevention bundle element. The horizontal axis indicates year/month.

The performance measures of SSI prevention bundle were proposed as the quality indicators of a quality improvement approach to reduce SSI rates. The improvements of monthly performance measures are shown in (Figure 4), including the following: a. the rates of pre-operative showers increased from 26.5% in 2019 to 90.5% in 2020; b. the rates of hair removal by clippers increased from 9.3% to 94.6%; c. the rates of maintaining preoperative blood glucose <180 mL/dL increased from 70.7% to 92.1%; d. the normothermia rates increased from 40.5% to 81.7%; e. the rates of appropriate prophylactic antibiotic administration were 98.7% in 2019 and 98.2% in 2020. All the targets of the five performance measures have been achieved.

The environmental cleaning was supervised and audited by managers of the general affairs office. To improve the cleaning practice, educational programs for appropriate cleaning procedures were provided for cleaning staff. The air system and clean traffic zones in the operating theater were well maintained. An audit protocol was implemented for environmental cleaning.

The antimicrobial stewardship program was strengthened to promote appropriate antimicrobial use, prevent the emergence of antimicrobial resistance, and finally result in better surgical outcomes. We developed an electronic surveillance and alert system for identifying patients receiving inappropriate antimicrobial therapy and notifying the physicians.

**Figure 5:**
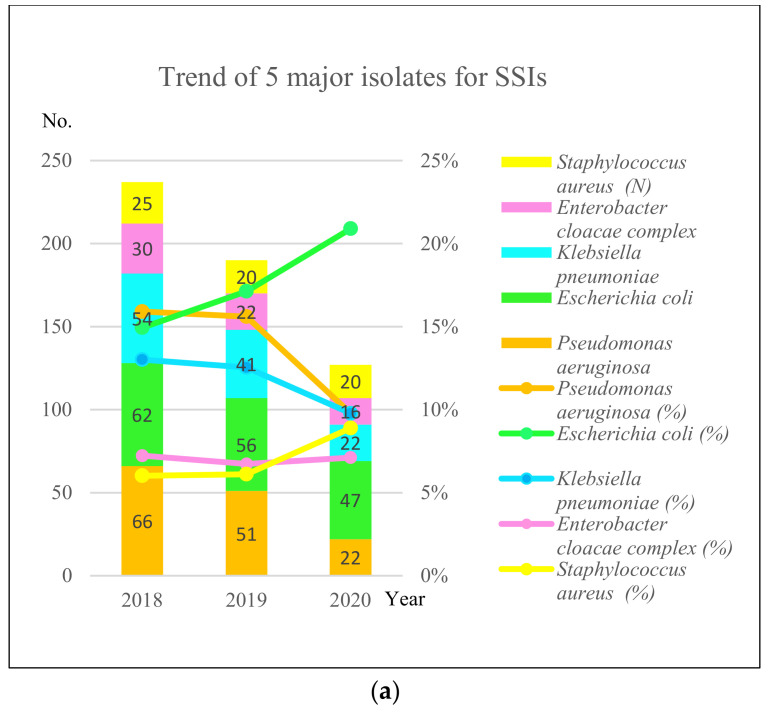

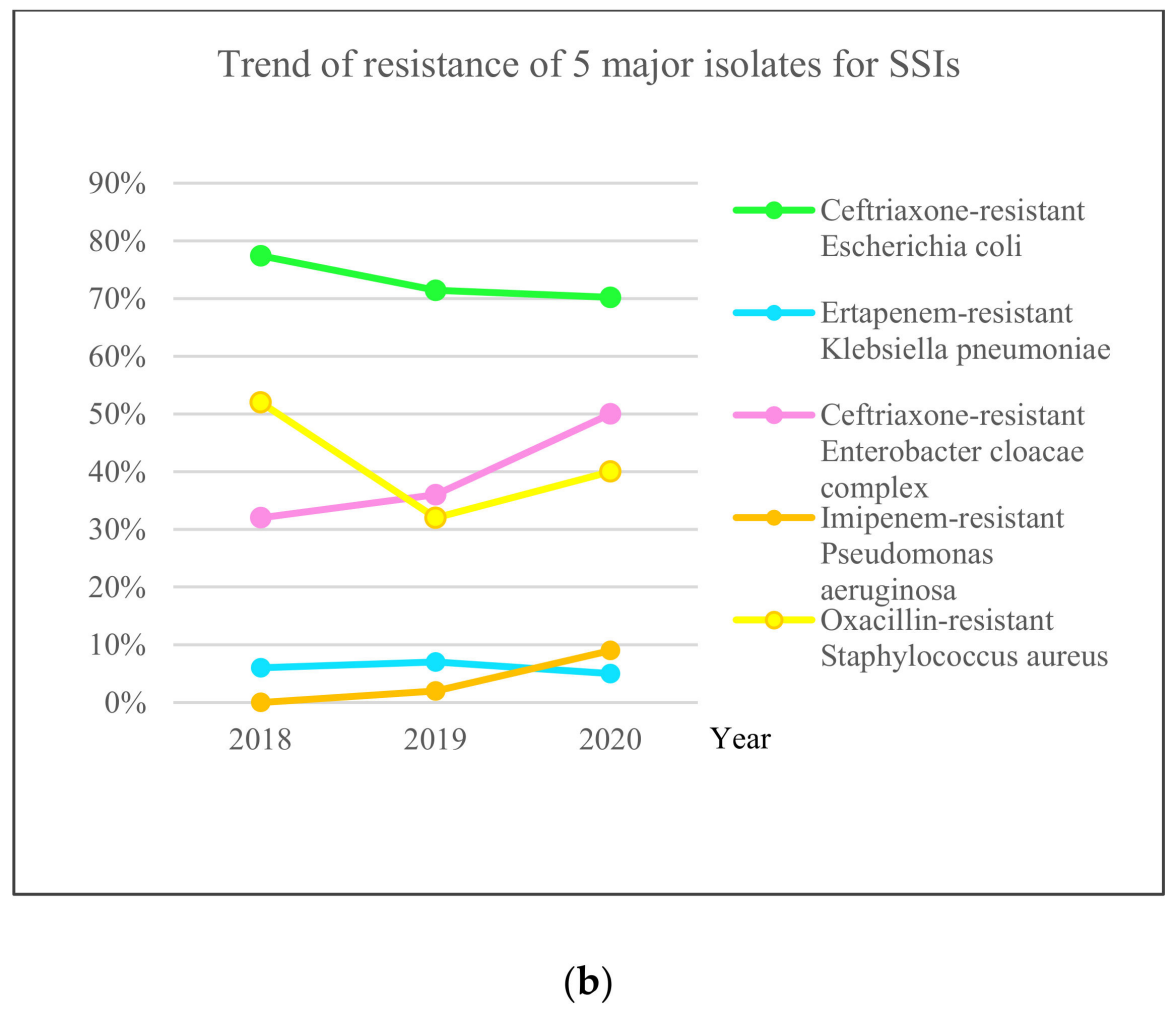
(**a**) Trend of five major isolates for SSIs. The left vertical axis indicates the number of isolates. The right vertical axis indicates the percentage of isolates. The horizontal axis indicates the year. The numbers in the color bars indicate numbers of isolates. Isolate (%) indicates percentage line of the isolate. (**b**) Trend of resistance of 5 major pathogens for SSIs. The vertical axis indicates the percentage of resistance. The horizontal axis indicates the year.

The trend of five major isolates of SSIs is shown in Figure 5a. The total numbers of isolates associated with SSIs were 415, 327, and 225 in 2018, 2019, and 2020, respectively. The most common isolates of SSIs were *Escherichia coli*, *Pseudomonas aeruginosa*, *Klebsiella pneumoniae*, *Enterobacter cloacae* complex, and *Staphylococcus aureus*. The numbers of these five major isolates are shown in the bars in Figure 5a. The numbers of *E. coli* isolates decreased; they were 62, 56, and 47 in 2018, 2019, and 2020, respectively. The numbers of *P. aeruginosa* isolates decreased; they were 66, 51, and 22 in 2018, 2019, and 2020, respectively. The percentages of *P. aeruginosa* among all isolates from SSIs in a year were 16%, 16%, and 10% in 2018, 2019, and 2020, respectively. Similarly, the percentages of *E. coli* were 15%, 17%, and 21%, respectively. The percentages of *K. pneumoniae* were 13%, 13%, and 10%, respectively. The percentages of *E. cloacae* complex were 13%, 13%, and 10%, respectively. The percentages of *S. aureus* were 6%, 6%, and 9%, respectively.

The trend of resistance of the major five isolates for SSIs is shown in Figure 5b. The ceftriaxone resistance rates of *E. coli* isolates were 77%, 71%, and 70% in 2018, 2019, and 2020, respectively. The ertapenem resistance rates of *K. pneumoniae* isolates were 6%, 7%, and 5% in 2018, 2019, and 2020, respectively. The ceftriaxone resistance rates of *E. cloacae* complex isolates were 32%, 36%, and 50% in 2018, 2019, and 2020, respectively. The imipenem resistance rates of *P. aeruginosa* isolates were 0%, 2%, and 9% in 2018, 2019, and 2020, respectively. The oxacillin resistance rates of *S. aureus* isolates were 52%, 32%, and 40% in 2018, 2019, and 2020, respectively.

**Figure 6:**
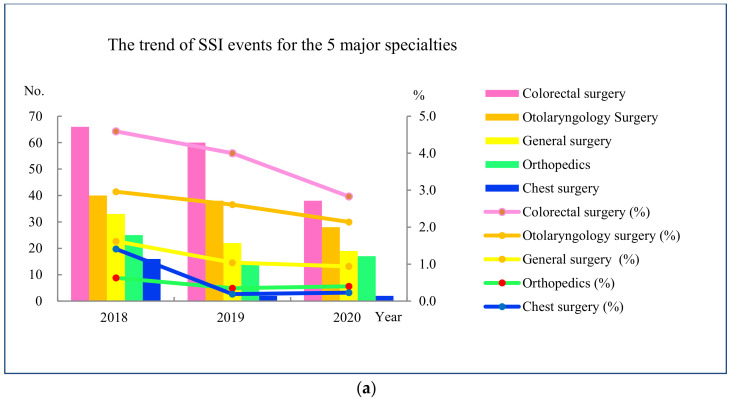

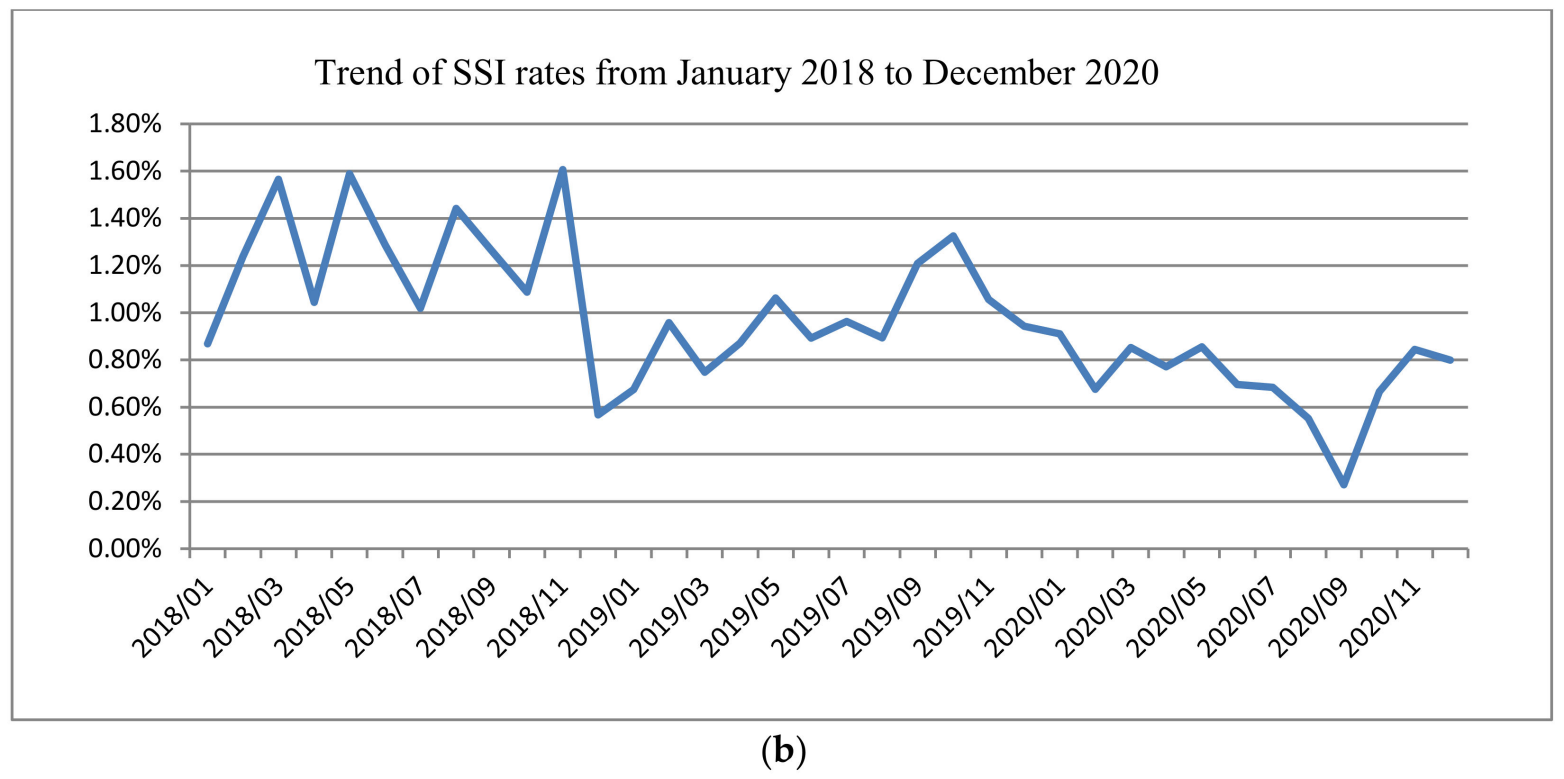
(**a**) The trend of SSI events in the 5 major specialties. The left vertical axis indicates the number of SSI events. The right vertical axis indicates the SSI rate. The specialty (%) indicates the SSI rate in the specialty. The horizontal axis indicates the year. (**b**) Trend of SSI rates from January 2018 to December 2020. The vertical axis indicates SSI rate. The horizontal axis indicates year/month.

The SSI rates were more common for the five major specialties, including the divisions of Colorectal Surgery, Otolaryngology Surgery, General Surgery, Orthopedics, and Chest Surgery (shown in Figure 6a). They had to report the identified defects, improvement strategies, and outcomes at the Infection Control Committee quarterly if the SSI rates rise.

The trend of SSI events is shown in Figure 6a. With the improvement processes, the SSI rates for colorectal surgery decreased from 4.6% (66 events in 1437 surgical operations) in 2018 to 2.8% (38 SSI events in 1342 surgical operations) in 2020. The SSI rates for otolaryngology surgery decreased from 3.0% (40 SSI events in 1352 surgical operations) in 2018 to 2.1% (28 SSI events in 1309 surgical operations) in 2020. The SSI rates for general surgery decreased from 1.6% (33 SSI events 2032 surgical operations) in 2018 to 0.9% (19 SSI events in 2020 surgical operations) in 2020. The SSI rates for orthopedic surgery decreased from 0.6% (25 SSI events in 3956 surgical operations) in 2018 to 0.4% (17 SSI events in 4215 surgical operations) in 2020. The SSI rates for chest surgery decreased from 1.4% (16 SSI events in 1135 surgical operations) in 2018 to 0.2% (2 SSI events in 884 surgical operations) in 2020.

### 3.5. Control

The average rates of performance measures in the pre-intervention, during intervention, and post-intervention period are shown in Table 3. The number and percentage of patients enrolled in each element of care bundle were analyzed. Chi-squared tests revealed significant improvements among the periods of pre-intervention, during intervention, and post-intervention (*p* < 0.001), except appropriate prophylactic antibiotic administration (*p* = 0.362). Because the rates of appropriate prophylactic antibiotic administration had reached 98.6% in the pre-intervention period, there was no significant change as compared with those in the post-intervention period. The rates of pre-operative shower increased from 3.1% for the pre-intervention period in 2019 to 57.4% for the post-intervention period in 2020. The rates of hair removal by clipper increased from 8.5% to 73.7%. The rates of appropriate prophylactic antibiotic administration were 98.6% in 2019 and 98.3% in 2020. The rates of maintaining blood glucose <180 mL/dL on the pre-operative day increased from 71.7% to 89.9%, and increased from 17.3% to 68.2% on postoperative day 1. The rates of maintaining normothermia increased from 55.2% to 74.4%. This study has achieved a significant 22.2% decline in SSI rates from 0.9% (89 SSI events in 10,297 surgical operations) for the pre-intervention period in 2019 to 0.7% (146 SSI events in 20,653 surgical operations) for the post-intervention period in 2020 (*p* = 0.004) (shown in Table 3). The trend of SSI rates from January 2018 to December 2020 is shown in Figure 6b. The SSI rates decreased significantly.

## 4. Discussion

The traditional quality-improvement strategies focus on the performance of individual surgeons and their errors retrospectively. Many errors cannot be prevented effectively, because the true problem is a lack of understanding of the system defects in the whole perioperative process [15]. For example, if the surgeons only focus on the surgical techniques and they don’t implement strategies to correct the risk factors of SSI and systemic defects in the surgical procedures, the SSIs cannot be adequately prevented. The DMAIC method focuses on a comprehensive systemic strategy for cyclic improvement processes, including leadership to change, organizing a multidisciplinary team to analyze the process defects, and implementation of interventions to eliminate the systemic process defects, and sustain the improvement. Therefore, the multimodal Six Sigma DMAIC method can work better than traditional quality-improvement strategies (i.e., preventive care bundle alone) to achieve the goal of performance improvement and reduce the rates of SSI [16,28,29].

During the project, the multidisciplinary prevention team developed policies and improvement processes for the reduction of SSIs. The dean and hospital administrators approved and enforced the policies and improvement processes, and supported the multidisciplinary prevention team in implementing them. The dean and hospital administrators provided the necessary resources for performance improvement. The Infection Control Committee run the contest for infection control measures, including hand hygiene, use of personal protective equipment, clean and disinfected environmental surfaces, isolation and cohort care, and sharps’ safety, etc. The Committee reported the review of the outcomes of the DMAIC improvement processes to the dean. The dean rewarded their successes in infection control and reduction of SSI rates every half year. The role of leadership is crucial to ensure that policies are followed for the effective interventions and best practice to reduce SSI risk events for patients and staff.

Using the DMAIC method, the multidisciplinary teams can identify and correct multiple modifiable defects across the surgical process [29]. For example, the endocrinologist team members play an important role in the perioperative glycemic control. Perioperative dys-glycemia is a risk factor not only for surgical site infections, but also increases risks of re-operation, poor prognosis, and re-hospitalization. Several clinical guidelines recommended target blood glucose level is 140–180 mg/dL for non-critical and critically ill inpatients [3,6]. To meet the optimal target, the Division of Endocrinology and Metabolism of our hospital established an outpatient department for scheduled surgical patients with poor preoperative glycemic control (glucose level higher than 180 mg/dL or HbA1C >9%). To minimize the infection rate of hospitalized patients, the inpatient glycemic management program included the following four processes: (1) An electronic dashboard that analyzed and monitored the glucose data of all hospitalized patients; (2) A glycemic management information system that can send warning messages to surgeons daily; (3) An endocrinologist team provides remote glycemic management recommendations; (4) Timely warnings and recommendations for the prevention of hypoglycemia [30,31].

Normothermia minimizes the risk of SSIs as well as other infections and cardiovascular events, postoperatively [27]. Maintaining perioperative normothermia is considered important in perioperative care and is recommended in guidelines for prevention of SSIs. However, some surgical team members did not recognize perioperative hypothermia as a risk of SSIs before this project. We have made healthcare staff aware of the risk of perioperative hypothermia through education during the intervention period. The performance of maintaining perioperative normothermia has improved by focusing on monitoring the temperature closely and the implementation of warming systems during the surgery. The patient would be kept at the normothermia status with blankets and heating lamps postoperatively.

The hospital surfaces surrounding patients are usually contaminated by multidrug-resistant organisms and thereby increase the risk of colonization, transmission, and infection [14,32]. HAIs can be prevented by enhanced environmental cleaning [33,34]. However, environmental cleaning efforts in hospitals are often not sufficient; thereby, microbial contamination will be present on hospital surfaces [35]. We implemented an audit project for the improvement of environmental cleaning by visual inspection and adenosine triphosphate bioluminescence tests during the period from 2019 to 2020. For environmental monitoring, we provided the audit reports to the managers of general affairs office who supervise the cleaning work, and got their feedback on the improvement of environmental cleaning practice. In our published article in 2021, the audit score of environmental cleaning by visual inspection was 87.5%. Continuous improvement on environmental cleaning still needs to be strengthened [32].

In this study, the resistance rates of isolates were calculated at the dates of SSI events reported. Although the carbapenem resistance rates of Enterobacteriaceae (e.g., *E. coli* and *K. pneumoniae*) and *P. aeruginosa* were lower than 10% at the dates of SSI events, carbapenem-resistant isolates could be selected during prolonged treatment with carbapenem for ceftriaxone-resistant Enterobacteriaceae. The oxacillin-resistant rates of *S. aureus* appeared to be decreasing from 52% in 2018 to 40% in 2020.

HAIs caused by ESBL-producing Enterobacteriaceae have led to the extensive use of carbapenems for treatment. Consequently, the emergence of carbapenem-resistant Enterobacteriaceae has become a more serious threat [36]. A laboratory-based strategy is an important part of antimicrobial stewardship program. The rapid and accurate diagnosis of antimicrobial susceptibility is necessary to optimize the therapeutic strategy for patients with infections caused by multi-drug resistant microorganisms [37]. The gold standards of antimicrobial susceptibility testing are based on disk diffusion method or minimum inhibitory concentration test, according to the guidelines standardized by the Clinical Laboratory Standards Institutes of USA or the European committee on Antimicrobial Susceptibility Testing. We have used MALDI-TOF MS system for rapid identification within 1 h after 18–24 h of incubation. We also have used the VITEK-2 system (bioMérieux) for susceptibility tests. Following 18–24 h of incubation, the susceptibility reports are available within 18 h. The MALDI-TOF MS system is more time-saving and cost-effective than the VITEK-2 system for identification of microorganisms [38].

Physicians usually will review the culture and susceptibility report to ensure the appropriate antibiotic use. However, if there is no automatic and efficient notification, the physicians may not review the culture and susceptibility immediately when it is reported. We have developed an electronic surveillance and alert system for identifying patients receiving inappropriate antimicrobial therapy since 2018. An electronic surveillance system can identify patients whose antibiotic therapy does not match the reported microbiologic susceptibilities, thereby automatically sending a “bug-antibiotic mismatch alert” message to the physician to ensure the appropriate antibiotic use. Furthermore, with the audit and feedback system, the adherence to guideline and infection rates can be improved [39]. However, auditing the performance measures by manual review of the medical records is labor-intensive and time-consuming. The machine learning techniques can provide accurate and efficient models for audit of the performance measures; for example, The machine learning techniques could be applied for the audit of appropriate prophylactic antimicrobial use [40]. The algorithms of machine learning techniques are more efficient in execution time than manual review.

During the control phase, a control plan was developed and implemented by the investigators to monitor and sustain the improvement. The efficacy and efficiency of the implemented improvement measures (education program for healthcare staff, providing feedback on SSI information to surgeons, adoption of standard clinical procedures and integration of change to clinical pathways) were continuously monitored. We also provided feedback on the efficacy and efficiency of the implemented improvement measures to the surgeons [24].

This study has achieved a significant 22.2% decline in SSI rates from 0.9% for the pre-intervention period in 2019 to 0.7% for the post-intervention period in 2020 (*p* = 0.004). The success is attributed to the multidisciplinary teams identifying multiple modifiable defects across the surgical process. In this project, many of the barriers to the improvement of performance measures arose from the lack of surgeon-driven leadership [24]. Leadership has played an important role in the success of reducing the SSI rates by the DMAIC improvement processes. The senior leaders have been asked to promote the individual accountability more actively, to work together with staff on a more comprehensive action plan. By education programs, the healthcare staff members are encouraged to create a safety culture by identifying and solving the problems, and then changing the procedures as necessary and integrating the change for improvement to normal work process [24]. This study has demonstrated that Six Sigma DMAIC is an effective approach in reducing the SSI rates.

There were limitations in this study. First, the evidence-based performance measures in this study are highly recommended by most guidelines and should be recommended for all surgical procedures [1,3,6]. However, the risk factors of SSI are complex and multifactorial [3,6,12], and this study did not analyze all the perioperative risk factors. Second, a meta-analysis has indicated that the effect of perioperative care bundles for the prevention of SSIs is inconsistent across randomized control trials [12]. No strong evidence for the characteristics of effective preventive care bundles was identified. Larger bundles were not associated with a better effect, but the better effect may be achieved if the care bundle contains a high proportion of evidence-based interventions [12]. Although we have achieved a success of the limited performance measures in this study, it is necessary for the societies of surgery and infection prevention to identify other effective measures that contribute to SSI prevention and outcome performance. A collaborative effort by the surgical societies will be needed to increase adherence to evidence-based SSI prevention practices and achieve the goal of SSI reduction [41].

## 5. Conclusions

Application of the Six Sigma DMAIC approach has been demonstrated to be an effective improvement approach in reducing the SSI rates in this study. Following strengthening leadership and empowering a multidisciplinary SSI prevention team, the DMAIC cycles were applied, including the five phases: define the appropriate goal, measure the performance data, analyze the possible causes of deviation from standard procedures, implement the improvement strategies and sustain the improvement. The performance measures of SSI prevention bundle were proposed as the quality indicators of quality improvement approach to reduce SSI rates.

All the targets of prevention bundle elements have been achieved. For example, the rates of maintaining preoperative blood glucose <180 mL/dL increased from 70.7% to 92.1% and the normothermia rates increased from 40.5% to 81.7%. We have implemented an environmental monitoring strategy to prevent colonization and transmission of multi-drug resistant microorganism. The antimicrobial stewardship program was strengthened by developing an electronic surveillance and alert system for identifying patients receiving inappropriate antimicrobial therapy and notifying the physicians. We also used the rapid and accurate diagnostic testing systems (MALDI-TOF and VITEK-2) to identify multi-drug resistant bacteria and to provide appropriate antimicrobial therapy. As a result, the carbapenem resistance rates of Enterobacteriaceae and *P. aeruginosa* were lower than 10%. The oxacillin resistance rates of *S. aureus* decreased from 52% in 2018 to 40% in 2020. This project has achieved the goal of reduction of SSI rates by a significant 22.2% decline in SSI rates from 0.9% for the pre-intervention period in 2019 to 0.7% for the post-intervention period in 2020 (*p* = 0.004).

The DMAIC model may help hospital administrators and quality management personnel implement systemic strategies to significantly reduce SSI rates, and assist in sustaining the performance improvement. In the future study, we still have the opportunities and challenges of sustaining improvement, including education programs, promoting individual accountability, and implementing more evidence-based measures to prevent SSI.

## Figures and Tables

**Table 1:** The Six Sigma DMAIC project statement.

Project Title
Application of Six Sigma DMAIC for the reduction of surgical site infections
**Problem to be solved**
The hospital’s SSI rate ranked as the highest 75th percentile in Taiwan
**Goal**
Implement corrective measures to reduce the risk of SSIs. Reduce the SSI rates by 20% by the end of 2020
**SSI Prevention team members**
Physicians (diabetologists, anesthesiologists, surgeons, and infectious disease specialist), quality control practitioners, infection control practitioners, nurses, and information technology engineers
**Implementation**
This project was conducted through the five phases of DMAIC over a period of 24 months from January 2019 to December 2020
**Schedule**
Define January 2018 Measure January 2019 Analyze June 2019 Improve January 2020 Control December 2020

**Table 2:** Risk events associated with surgical site infections and solutions to reduce the SSI rates.

Risk Events	Solutions
Leadership support system to be strengthened	Establish a culture of safety with strong leadership support: Providing needed resources for performance improvement. Reviewing the performance measures for infection prevention regularly. Rewarding employee successes in improving performance and infection reduction. Organizing a multidisciplinary teamwork among surgical services and other support departments to implement strategy of improvement.
Lack of perioperative risk assessment system	Analyzing the major risk factors associated with SSI to develop an infection prevention plan and to implement strategies to improve performance of procedures.
Perioperative protocols to be standardized	Application of the evidence-based interventions to set up standard perioperative protocols for specific procedures. Application of a clinical pathway information system to improve the process.
Lack of healthcare information feedback system	Providing feedback on SSI information and performance measures to the surgeons.
Lack of audit system for environmental cleaning	Providing education for staff on appropriate cleaning procedures. Maintenance of air systems and clean traffic zones in the operating theater. Implementation of an audit protocol for environmental cleaning.
Antimicrobial stewardship program to be strengthened	Developing an electronic surveillance and alert system for identifying patients receiving inappropriate antimicrobial therapy and notifying the physicians. Auditing the appropriate use of prophylactic antibiotics.
Lack of education program for prevention of SSI	Providing an education program with the following contents: SSI surveillance. Improvement projects of perioperative process to reduce SSIs. Emphasis on the senior-driven leadership and individual accountability for safety culture.

**Table 3:** Comparison of the performance measures of care bundle and SSI rates. Pre-intervention: January 2019 to June 2019; during-intervention: July 2019 to December 2019; post-intervention: January 2020 to December 2020.

	Pre-Intervention	During-Intervention	Post-Intervention	*p* Value **
	N (%) *	**N (%)**	**N (%)**	
Preoperative shower				<0.001
No	9898 (96.9)	6791 (78.5)	6294 (42.6)	
Yes	312 (3.1)	1862 (21.5)	8478 (57.4)	
Hair removal by clipper				<0.001
No	1604 (91.5)	685 (77.9)	358 (26.3)	
Yes	149 (8.5)	194 (22.1)	1002 (73.7)	
Appropriate prophylactic antibiotic administration				0.362
No	122 (1.4)	166 (1.6)	309 (1.7)	
Yes	8438 (98.6)	10,058 (98.4)	18,380 (98.3)	
Glucose control, pre-operative				<0.001
No	2892 (28.3)	2070 (23.9)	1488 (10.1)	
Yes	7318 (71.7)	6583 (76.1)	13,284 (89.9)	
Glucose control, post-operative day 1				<0.001
No	8446 (82.7)	4964 (57.4)	4700 (31.8)	
Yes	1764 (17.3)	3689 (42.6)	10,072 (68.2)	
Glucose control, post-operative day 2				<0.001
No	8082 (87.0)	3991 (76.2)	3156 (54.2)	
Yes	1208 (13.0)	1248 (23.8)	2667 (45.8)	
Maintaining normothermia				<0.001
No	3880 (44.8)	5635 (55.2)	3783 (25.6)	
Yes	4773 (55.2)	4575 (44.8)	10,989 (74.4)	
SSI rates				0.004
No	10,208 (99.1)	11,163 (98.9)	20,507 (99.3)	
Yes	89 (0.9)	120 (1.1)	146 (0.7)	

* N indicates no. of patients enrolled in the element of care bundle, % indicates the percentage of patients among the element of care bundle. ** *p* value of Chi-squared test.

## Data Availability

The data presented in this study are available on request from the corresponding author.
